# Effects of Amino Acid Supplementation in Low-Protein Diets on Productive Performance, Digestive Function, and Intestinal Health of Laying Hens

**DOI:** 10.3390/ani16081232

**Published:** 2026-04-17

**Authors:** Chongyang Zhang, Kangle Wu, Fang Wang, Shihang Yang, Jiayang Li, Meizhu Xie, Yulong Yin, Kang Yao

**Affiliations:** 1 Institute of Subtropical Agriculture, Chinese Academy of Sciences, Changsha 410125, China; chongyangz7931@163.com (C.Z.); yinyulong@isa.ac.cn (Y.Y.); 2 Hunan Collaborative Innovation Center for Utilization of Botanical Functional Ingredients, College of Animal Science and Technology, Hunan Agricultural University, Changsha 410127, China

**Keywords:** laying hen, low-protein diet, amino acid supplementation, production performance, total tract retention

## Abstract

This study aimed to evaluate the compensatory effects of amino acid (AA) supplementation on laying hens fed a low-protein diet, focusing on production performance, egg quality, blood immunity, total tract retention, and intestinal health. A total of 180 Hy-Line Brown laying hens (35 weeks old) were randomly allocated to three dietary treatments with 12 replicates (5 hens/replicate): (1) control diet (15.90% crude protein, CON), (2) reduced-protein diet (15.20% crude protein, NG), and (3) reduced-protein diet supplemented with 400 g/t methionine, 400 g/t lysine, and 300 g/t threonine (15.20% crude protein, LAA). Over the 12-week experimental period, production parameters were monitored weekly. The results showed that compared to the NG group, AA supplementation significantly increased laying rates during weeks 1–4 (*p* < 0.05) and reduced the feed-to-egg ratio during weeks 9–12 (*p* < 0.05), with a 3.10% decrease in feed conversion ratio (FCR). By week 12, the NG group exhibited lower Haugh unit values than both the CON and LAA groups (*p* < 0.05), indicating improved egg quality with AA supplementation. Serum analysis revealed elevated immunoglobulin Y levels and upregulated expression of the anti-inflammatory cytokine IL-10 in the LAA group (*p* < 0.05). Total tract retention of crude protein (CP) and phosphorus (P) was significantly enhanced in the LAA group compared to CON and NG groups (*p* < 0.05). However, in terms of cecal microbiota, no significant differences were observed among the experimental groups. These findings demonstrate that AA supplementation alleviates the adverse effects of low-protein diets by enhancing nutrient utilization and immune modulation, thereby improving productive performance, egg quality, and total tract retention in laying hens.

## Simple Summary

Reducing dietary protein in layer hen feeds is a potential strategy for lowering costs and environmental impact, but it often compromises productivity and health. This study investigated whether supplementing such low-protein diets with essential amino acids could mitigate these negative effects. We fed 180 laying hens (35 weeks of age) one of three diets: a standard protein diet, a low-protein diet, or a low-protein diet fortified with amino acids. Over 12 weeks, we found that amino acid supplementation successfully counteracted the drawbacks of the low-protein diet. It restored laying rates, improved egg quality (specifically the Haugh unit, a measure of egg white quality), and enhanced the birds’ immune response. Furthermore, hens fed the amino acid-fortified diet showed better overall utilization of protein and phosphorus. These results confirm that targeted amino acid supplementation is a viable, cost-effective, and sustainable approach to using low-protein diets in layer operations, maintaining performance while supporting bird health and nutrient efficiency.

## 1. Introduction

Low-protein diets, characterized by reduced crude protein (CP) content supplemented with targeted amino acids (AAs), have emerged as a significant strategy in layer production due to their economic and environmental benefits, such as lower feed costs and reduced nitrogen emissions. Strategic reduction in dietary CP by 2–4%, when combined with essential AA supplementation, has been shown to maintain production performance while decreasing nitrogen excretion and alleviating gastrointestinal issues [1,2]. This approach allows more precise alignment of dietary formulations with nutritional requirements, thereby enhancing protein utilization and minimizing environmental impact [3,4].

In corn–soybean meal-based poultry diets, methionine (Met), lysine (Lys), and threonine (Thr) are typically the first limiting amino acids due to the bird’s limited ability to synthesize them endogenously [5,6]. These AAs play critical roles beyond basic nutrition, influencing protein synthesis, immune response, antioxidant capacity, and gut health. Met, as the first limiting AA in corn–soybean diets, is essential for protein structure, skeletal development, and redox homeostasis [7,8]. However, improper levels of Met may adversely affect feed intake and nutrient absorption [9]. Lysine, the second limiting AA, significantly influences growth performance, carcass traits, and immune function [10]. In laying hens, adequate Lys supports egg production efficiency and egg mass [11]. Threonine is particularly important for intestinal barrier function and immunity, serving as a key component in mucin (MUC2) and antibody synthesis [12,13]. Manifestations of Thr deficiency vary between poultry types: broilers often show impaired growth, whereas layers experience reduced egg production and quality [14,15]. Its role is especially critical in low-CP diets for maintaining digestive enzyme secretion and gut microbiota balance [16]. Although some studies report positive effects of Thr supplementation on layer performance [17], others have found no significant benefits [18].

Although low-protein (LP) diets have been extensively studied in broilers, the underlying mechanisms in laying hens remain inadequately understood. Existing research shows inconsistent responses to crude protein reduction, with some studies reporting maintained performance [19,20], while others document reduced productivity [21,22]. These inconsistencies may arise from differences in amino acid balancing strategies, environmental conditions, or genetic backgrounds. Moreover, the relationship between LP diets, total tract nutrient retention, and intestinal health has not been sufficiently examined. Although threonine supplementation has produced variable effects on layer performance [17], its potential role in alleviating microbiota dysbiosis induced by low-CP diets requires further exploration. This study aims to address the research gap concerning the supplementation of the three most limiting amino acids during the peak laying period in hens. In contrast to previous studies that reduced dietary crude protein by 2–4%, the current trial implemented a more modest reduction of only 0.7%. Therefore, this research systematically investigates the effects of LP diets supplemented with limiting amino acids on laying performance, nutrient digestibility, and intestinal health in laying hens. By elucidating key aspects of nutrient utilization and gut function under protein-restricted nutrition, our results seek to support the adoption of sustainable feeding strategies in commercial egg production.

## 2. Materials and Methods

### 2.1. Ethical Approval

All experimental procedures involving animals were reviewed and approved by the Institutional Animal Care and Use Committee of the Institute of Subtropical Agriculture, Chinese Academy of Sciences (Changsha, China).

### 2.2. Experimental Design, Animals, and Diets

This experiment was conducted at the Hunan Zhongsheng Farming and Husbandry Layer Farm. A total of 180 Hy-Line Brown laying hens, aged 35 weeks, were randomly allocated to three dietary treatments in a completely randomized design. Each treatment consisted of 12 replicates, with 5 birds per replicate (housed in one cage). The experiment spanned a 12-week period. The dietary treatments included: (1) a basal diet (CON) with standard crude protein content, (2) a reduced-protein diet (NG), and (3) a reduced-protein diet supplemented with 400 g/t methionine, 400 g/t lysine, and 300 g/t threonine (LAA). Detailed compositions and nutrient levels of the experimental diets are provided in Table 1. All diets were formulated in mash form and provided ad libitum throughout the trial.

Five hens were housed per stainless steel cage (40 cm W × 45 cm L × 45 cm H). This configuration provided a stocking density of 450 cm^2^ per hen. Environmental conditions were strictly controlled: a photoperiod of 16 h light:8 h dark (combining natural and artificial illumination) was maintained, while ambient temperature was regulated between 18 °C and 24 °C. The trial included a 2-week adaptation phase followed by a 12-week experimental period for data collection.

### 2.3. Evaluation of Production Performance

Feed intake, egg mass, and average egg weight were recorded daily and summarized weekly. Hen-day egg production, average daily feed intake (ADFI), laying rate and feed conversion ratio (FCR, calculated as total feed intake divided by total egg mass) were determined over the 12-week experimental period. Flock health was monitored daily, with mortality and clinical abnormalities documented systematically.

### 2.4. Egg Quality Measurements

At the end of week 12, 108 eggs (3 eggs per replicate × 36 replicates) were collected for analysis, excluding abnormal specimens (pee wee, soft-shelled, cracked, or double-yolked eggs). Egg weight (EW) was measured using a digital balance (ATY224, Shimadzu, Kyoto, Japan, ±0.01 g). Fresh eggs were broken onto a flat surface, and albumen height (AH) was quantified with a TSS QCD meter (Technical Services and Supplies Ltd., York, UK). Haugh units (HU) were calculated using the formula: HU = 100 × log(AH(mm) + 7.57 − 1.7 × EW(g) 0.37). Yolks were separated by removing chalazae with forceps, rolled on absorbent paper to remove residual albumen, and weighed to determine yolk percentage (YP(%) = yolk weight(g)/EW(g) × 100). Egg shape index (ESI = maximum width(mm)/length(mm) × 100) was calculated. Eggshells were rinsed, air-dried at 25 °C for 48 h, and evaluated for shell strength (SS) using a texture analyzer (TA.XT PlusC, SMS, Godalming, UK). Shell thickness (ST) was measured at three positions (equator, blunt end, sharp end) with a thickness gauge (NFN-380, FHK, Tokyo, Japan), and the average value was recorded.

### 2.5. Serum Biochemical Analysis

Blood was drawn from the wing veins of selected hens (n = 6) into EDTA anticoagulant tubes. Serum was separated by centrifugation (3000× *g*, 15 min, 4 °C) and stored at −20 °C. Immunoglobulins (IgA: ANG-E32004C, 10–600 ng/mL; IgG: ANG-E32005C, 0.1875–11.25 mg/mL; IgY: ANG-E32209C, 0.0625–3.75 ng/mL) and cytokines (IL-10: ANG-E32011C, 1–60 ng/L; IL-6: ANG-E32013C, 18.75–1125 pg/mL; TNF-α: ANG-E32030C, 1.25–75 ng/L) in serum were quantified using ELISA kits (Nanjing Angle Gene Bioengineering Co., Ltd., Nanjing, China). All assays exhibited intra- and inter-assay coefficients of variation below 9% and 15%, respectively. Serum samples were diluted 5–10× prior to analysis, following manufacturer protocols.

### 2.6. Analysis of Total Tract Nutrient Digestibility

To assess the impact of dietary restricted amino acid supplementation on nutrient retention, a retention trial was conducted during the final 5 days of the experiment. Six replicates were randomly selected, and total excretions from each replicate were collected quantitatively. Following this, excreta samples were collected quantitatively for five consecutive days. During the collection period, feed and water were supplied ad libitum. The total feed intake was recorded for each replicate, and fresh excreta were collected twice daily (morning and evening). The samples were pooled per replicate, weighed, freeze-dried, ground to a fine powder, and stored in airtight containers for chemical analysis. The proximate composition of both feed and excreta samples was analyzed following the standard procedures of the Association of Official Analytical Chemists. Parameters analyzed included crude protein (CP), Crude ash (Ash), Calcium (Ca) and Phosphorus (P). Acid-insoluble ash was employed as an endogenous marker to calculate retention coefficients using the formula:

Total tract retention(%) = (1 − (Nutrient content in excreta(%))/(Nutrient content in feed(%)) × “Marker content in feed” (%)/“Marker content in excreta” (%)) × 100

### 2.7. Gut Sample Collection and 16S rRNA Sequencing

Cecal contents (n = 6, the six samples per group were collected from one randomly selected hen per every two replicates) were aseptically collected into sterile tubes, flash-frozen in liquid nitrogen, and stored at −80 °C until DNA extraction. For in-depth analysis of the cecal microbiota, high-throughput sequencing was performed. Microbial genomic DNA was first extracted from cecal contents using the QIAamp DNA Stool Mini Kit (Qiagen, Germantown, MD, USA) according to the manufacturer’s instructions. DNA purity and concentration were assessed with a NanoDrop ND-1000 spectrophotometer (Thermo Fisher Scientific, Waltham, MA, USA).

The V3 hypervariable region of the 16S rRNA gene was amplified from the extracted DNA using barcoded fusion primers 338F (5′-ACTCCTRCGGGAGGCAGCAG-3′) and 806R (5′-GGACTACHVGGGTWTCTAAT-3′). PCR was carried out under the following conditions: initial denaturation at 94 °C for 3 min, followed by 35 cycles of denaturation, annealing, and extension, with a final extension step. The PCR products were separated on a 2% agarose gel and purified using the Axyprep DNA Gel Extraction Kit (Axygen Scientific Inc., Union City, CA, USA). After purification, sequencing adapters and poly(A) tails were attached to construct the library. Finally, the library was sequenced on an Illumina MiSeq platform by RiboBio Co., Ltd. (Guangzhou, China).

Raw tags were filtered to obtain clean and high-quality tags, chimera sequences were removed, and sequences with 97% similarity were clustered into operational taxonomic units (OTU) by QIIME2. The alpha diversity indices and beta diversity of the cecal microbiota were calculated withQIIME2 and displayed with R software (version 4.3.1; R Core Team, Vienna, Austria, 2023). Beta diversity was determined using the Bray–Curtis index and visualized as principal coordinate analysis (PCoA) and non-metric multidimensional scaling (NMDS) plots.

### 2.8. Statistical Analysis

All data were analyzed using a one-way analysis of variance (ANOVA, SPSS 26.0 for Windows). Prior to statistical analysis, data normality was tested using the Shapiro–Wilk test, and homogeneity of variances was evaluated using Levene’s test. For any response variable that did not meet the normality assumption, logarithmic transformations were applied. Mean comparisons between treatments were conducted using Tukey’s honest significant difference (HSD) test to identify significant differences. Values were considered statistically different at *p* < 0.05.

## 3. Results and Analysis

### 3.1. Results

#### 3.1.1. Production Performance

The effects of low-protein diets supplemented with amino acids on laying performance are presented in Table 2. During weeks 1–4, the laying rate in the LAA group (low-protein + amino acid supplementation) was significantly higher than that in the NG group (no amino acid supplementation; *p* < 0.05). Throughout the experimental period, the laying rates of the CON (control, standard protein) and LAA groups were significantly higher than that of the NG group (*p* < 0.05). However, no significant differences were observed among groups in average daily feed intake (ADFI), average egg weight, or feed conversion ratio (FCR; *p* > 0.05).

#### 3.1.2. Egg Quality

As shown in Table 3, eggs from the CON and LAA groups exhibited significantly higher Haugh units compared to the NG group (*p* < 0.001), indicating improved albumen quality with amino acid supplementation. No significant differences were detected in egg weight, eggshell thickness, eggshell strength, egg shape index, or yolk ratio across groups (*p* > 0.05).

#### 3.1.3. Serum Parameter Analysis

Amino acid supplementation significantly increased serum immunoglobulin (IgY) and anti-inflammatory cytokine IL-10 levels compared to the CON and NG groups (*p* < 0.05; Table 4). In contrast, pro-inflammatory cytokines (IL-6 and TNF-α) remained unaffected (*p* > 0.05).

#### 3.1.4. Total Tract Retention of Nutrients

The LAA group demonstrated significantly higher total tract retention of crude protein (CP) compared to the CON and NG groups (*p* < 0.05; Table 5). Additionally, phosphorus total tract retention in the CON and NG groups was significantly lower than in the LAA group (*p* < 0.05). However, no significant differences were observed in ash, calcium (Ca) total tract retention among groups (*p* > 0.05).

#### 3.1.5. Diversity of Microbial Community in Laying Hens

**Figure 1:**
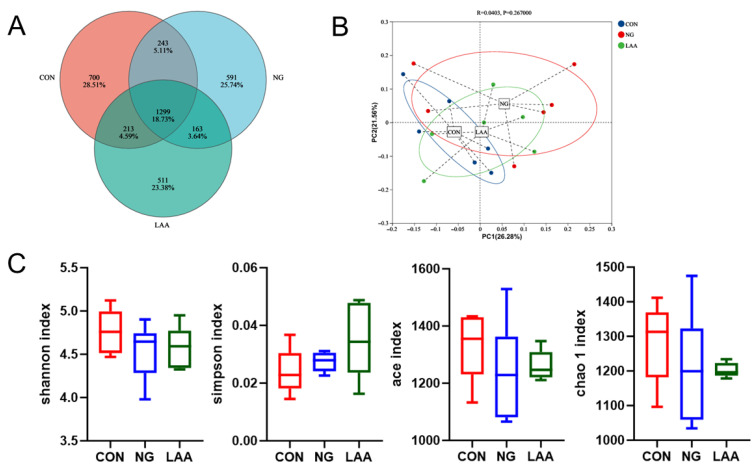
Microbial composition of cecal contents (n = 6). (**A**) Venn diagram of intestinal microflora OTUs. (**B**) The principal coordinate analysis (PCoA) of the cecum microbiota based on unweighted UniFrac metric. (**C**) Alpha diversity index analysis (Chao1 index; Shannon index; Simpson index; Ace index). CON: a basal diet, with standard crude protein content; NG: a reduced-protein diet; LAA: a reduced-protein diet supplemented with 400 g/t methionine, 400 g/t lysine, and 300 g/t threonine.

**Figure 2:**
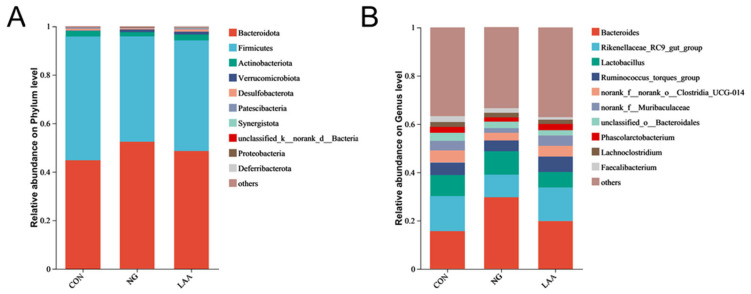
Relative abundance of species at the phylum level (**A**) and the genus-level species (**B**). The abundance is represented in terms of the percentage of the total effective bacterial sequences in the sample. The top 10 abound taxa are displayed. CON: a basal diet, with standard crude protein content; NG: a reduced-protein diet; LAA: a reduced-protein diet supplemented with 400 g/t methionine, 400 g/t lysine, and 300 g/t threonine.

**Figure 3:**
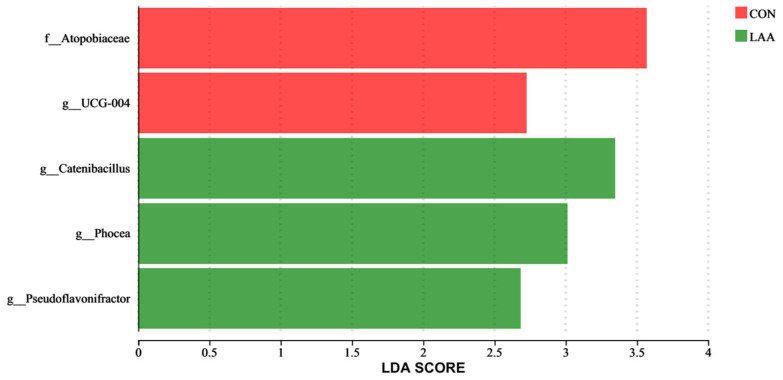
Linear discriminant analysis of effect size (LEfSe) analyses of cecum microbiota, histogram of the linear discriminant analysis (LDA) scores computed for differentially abundant bacterial taxa at the genus level between the CON, NG and LAA (LDA score > 2.5). n = 6.

A total of 3720 operational taxonomic units (OTUs) were shared across all groups, with 2455, 2296, and 2186 unique OTUs identified in the CON, NG, and LAA groups, respectively (Figure 1A). According to the principal coordinate analysis (PCoA), there were no significant differences in gut microbiota composition between the CON, NG, and LAA groups (Figure 1B). Alpha diversity indices (Chao1, Ace, Simpson and Shannon) showed no significant differences among groups (*p* > 0.05; Figure 1C). No significant differences were observed across groups in phylum-level (Figure 2A) or genus-level (Figure 2B) analyses. Furthermore, to identify the specific bacterial taxa characteristic of the different treatment groups, Linear Discriminant Analysis Effect Size (LEfSe) was performed. Using an LDA score threshold of >2.5, the analysis (Figure 3) revealed distinct microbial signatures between the CON, NG and LAA groups. The family f_Atopobiaceae and the genus g_UCG-004 were significantly enriched in the CON group. In contrast, the LAA group showed a marked enrichment in the genera g_Catenibacillus, g_Phocea, and g_Pseudoflavonifractor.

## 4. Discussion

Reducing dietary protein levels to 13–14% adversely affects layer performance unless synthetic amino acids (AAs) are promptly supplemented [22]. This observation is consistent with Liu et al.’s conclusion that AA supplementation in low-protein diets enhances productivity while reducing nitrogen emissions—a finding supported by multiple studies [20,23]. Our results also demonstrate that AA-supplemented low-protein diets significantly improve laying rates and reduce FCR during peak production. These benefits are likely attributed to two mechanisms: (1) compensation for limiting AAs, and (2) the rapid absorption kinetics of synthetic AAs, which may enhance physiological efficiency by optimizing glucose metabolism and hormonal regulation. However, the existing literature presents inconsistent findings on the effects of crude protein reduction with some studies reporting maintained performance under reduced CP levels [19,20], while others observe adverse effects compared to conventional protein levels [21,22]. Notably, our study detected no significant differences in daily feed intake or egg weight among treatment groups. These discrepancies may be influenced by factors such as genetic background, precision of dietary formulation, and housing environments. Alternatively, the absence of negative effects in our trial could be due to the relatively modest extent of CP reduction (0.7%) in the experimental diets relative to the control. This complexity highlights the need for context-specific nutritional strategies that consider breed-specific traits, management conditions, and production objectives. Our results indicate that supplementing limiting amino acids can fully compensate for a 0.7% reduction in dietary crude protein, not only maintaining baseline performance but also leading to improvements in certain efficiency metrics. These findings offer a viable model for enhancing commercial egg production and quality, thereby supporting the development of sustainable large-scale poultry operations.

The manipulation of layer diets to enhance egg quality remains a critical focus in poultry science. Dietary manipulation to improve egg quality continues to be a major research priority in poultry science. Although reduced CP diets offer potential environmental advantages, their effects on egg quality traits warrant careful assessment. In the present study, a 0.7% reduction in dietary CP from baseline levels, supplemented with limiting amino acids, did not significantly affect key egg quality attributes such as eggshell thickness, shell strength, or yolk ratio. This finding contrasts with reports that reducing dietary crude protein by 2% resulted in reduced albumen quality [24], implying that tailored amino acid supplementation may help counteract such negative effects. The mechanisms influencing egg quality in response to dietary protein appear to be multifactorial. Low-CP diets may downregulate the expression of amino acid transporters in intestinal tissue, thereby limiting systemic AA availability for protein synthesis in the egg [25,26]. Furthermore, deficiencies in essential AAs can promote catabolism of non-limiting AAs for use in non-productive metabolic pathways, ultimately inhibiting ovoprotein synthesis [27]. In this study, however, the dietary formulation—specifically designed to meet the amino acid requirements of Hy-Line Brown hens—likely averted such issues through precise LAA balancing. This result is consistent with Zhang [28], who maintained egg mass under AA-balanced low-CP conditions, in contrast to studies that observed production impairments when AA profiles were unbalanced [29]. Discrepancies among studies may arise from differences in the extent of CP reduction and the strategy of AA supplementation. For instance, whereas Parenteau et al. [24] observed deterioration in albumen quality with a 2% CP reduction, our more moderate reduction of 0.7%, accompanied by an optimized AA ratio, successfully preserved all egg quality parameters. These outcomes emphasize that gradual CP reduction combined with meticulous AA balancing is essential for maintaining both production efficiency and egg quality. Future studies should aim to establish breed-specific CP and AA thresholds and examine the long-term effects of various dietary regimens on albumen matrix formation.

The interaction between immunoglobulins and inflammatory mediators plays a crucial role in regulating immune competence and host defense mechanisms [30]. Immunoglobulin A (IgA) serves as a key component of mucosal immunity, IgM provides a rapid response to acute infections, and IgY acts as the primary circulating antibody in birds [31,32]. Meanwhile, cytokines such as TNF-α, along with both pro- and anti-inflammatory mediators, orchestrate inflammatory reactions, immune surveillance, and lymphocyte homeostasis [33,34]. These immune markers are closely tied to metabolic health, as studies have shown that deficiencies in dietary amino acids—particularly methionine (Met)—impair protein synthesis, reduce egg production efficiency, and alter serum biochemical profiles in poultry [35,36,37,38]. Notably, insufficient Met intake disrupts hepatic protein metabolism and weakens antioxidant capacity, amplifying systemic physiological stress [39]. Our results are consistent with growing evidence that optimized amino acid supplementation enhances humoral immunity in laying hens. The increased levels of serum IL-10 and IgY following supplementation with methionine, lysine, and threonine suggest enhanced anti-inflammatory regulation and antibody-mediated immune protection. This supports earlier findings that methionine supplementation improves metabolic function and immune responses in poultry [39]. Immunoglobulins, including IgA, IgM, and IgY, serve as essential elements of avian immunity, enabling broad antigen recognition and pathogen neutralization [40,41]. The current study expands on these insights by demonstrating that targeted amino acid supplementation can fine-tune specific immune parameters—a phenomenon also observed in broilers, where lysine and threonine improved mucosal immunity and antibody diversity [42,43]. The absence of significant changes in IgA, IgM, TNF-α, and IL-6 in our trial may reflect differential metabolic allocation of supplemented amino acids. This idea is supported by studies showing that laying hens prioritize reproductive protein synthesis over systemic immune activation during peak production, leading to tissue-specific amino acid utilization [36,37]. Moreover, stable pro-inflammatory cytokine levels indicate that the amino acid regimen maintained immune homeostasis without triggering excessive inflammation—a beneficial outcome for sustaining both productivity and disease resistance in commercial poultry operations. These findings underscore the importance of balanced amino acid nutrition in poultry diets, highlighting the distinct roles of methionine, lysine, and threonine in supporting protein metabolism and immune function. Future studies should explore the temporal dynamics of immune responses to amino acid supplementation and define optimal inclusion levels to simultaneously enhance productivity and immunocompetence.

Total tract nutrient retention is a critical indicator for assessing the efficiency of digestion and absorption of dietary components in livestock and poultry, with direct implications for production performance. In the present study, dietary supplementation with AAs significantly increased the total tract retention of CP and phosphorus in Hy-Line Brown laying hens. This finding is consistent with previous reports in broilers, where AA supplementation improved CP utilization efficiency [44,45]. The mechanism may involve two primary pathways: firstly, supplemental AAs may compensate for dietary limiting amino acids, optimizing AA balance and reducing the energy cost associated with protein turnover [46,47]. Secondly, protein-bound AAs must be hydrolyzed into absorbable small peptides and free AAs before intestinal absorption [48]. Supplemental AAs may enhance protease activity or alleviate competitive inhibition of peptide transporters, thereby improving the efficiency of protein hydrolysis. On the other hand, wheat bran was incorporated into the low-protein diet used in this trial. Although wheat bran is high in phosphorus, most of it is in the form of phytate-bound phosphorus, yet the total available phosphorus content was maintained at a consistent level. The results demonstrated that reducing dietary protein levels while supplementing with AAs improved phosphorus retention, even in the absence of phytase supplementation. This improvement may be attributed to a more balanced amino acid supply, which supported enhanced production performance and metabolic efficiency, thereby promoting phosphorus utilization for skeletal development, muscle growth, and eggshell formation, ultimately increasing phosphorus retention [49,50,51].

The intestinal microbiota plays a fundamental role in maintaining poultry health by influencing intestinal morphology, immune function, nutrient absorption, and growth performance [52]. This complex microbial community is influenced by multiple determinants, including host genetics [53], sexual dimorphism [54], developmental stage [55], dietary composition [56], environmental conditions [57], and health status [58]. Notably, dietary ingredients exert a stronger influence on the cecal microbiota than environmental factors, highlighting the critical role of nutrition in modulating microbial populations [59]. Recent studies have increasingly examined the dynamic interactions among dietary components—particularly metabolizable energy and crude protein—intestinal physiology, and microbial community structure [51,60,61,62,63]. Our analysis revealed no significant differences in α- and β-diversity indices among the experimental groups. This absence of divergence may be attributable to the limited sample size per group and the relatively modest reduction in dietary crude protein (0.7%). A larger cohort or a more substantial reduction in protein content might be necessary to elicit discernible shifts in microbial diversity. Consistent with prior findings [64], the cecal microbiota was predominantly composed of Bacteroidetes, Firmicutes, and Actinobacteria at the phylum level. The functional roles of these phyla are of particular interest: Firmicutes are involved in host energy metabolism, whereas Bacteroidetes facilitate polysaccharide degradation through glycoside hydrolases, producing volatile fatty acids [65]. LEfSe analysis (LDA > 2.5) further identified key bacterial genera enriched in the LAA group, including g_Catenibacillus, g_Phocea, and g_Pseudoflavonifractor. Notably, these genera are functionally linked to short-chain fatty acid (SCFA) metabolism and host energy regulation. g_Catenibacillus can deglycosylate flavonoids to produce metabolites with anti-inflammatory and antioxidant activities [66,67], while g_Pseudoflavonifractor is involved in butyrate production, which has been shown to serve as a source of nutrients for the intestinal epithelial cells and to promote intestinal epithelial cell growth [68,69]. Additionally, g_Phocea has been associated with host energy homeostasis and lipid metabolism [70]. Thus, the LAA diet appears to selectively promote a microbial community geared toward enhanced SCFA synthesis and metabolic efficiency, which may underpin the observed improvements in nutrient utilization. This subtle shift contrasts with more pronounced effects reported in studies implementing larger (2–4%) protein reductions, which may explain the stability of the core microbiota observed here. Together, these results indicate that a minor reduction in dietary crude protein (0.7%), when accompanied by essential amino acid supplementation, has limited impact on gut microbial composition. Maintaining microbial diversity is crucial, given its role in colonization resistance against pathogens. A balanced microbiota is essential not only for efficient nutrient utilization but also for supporting overall host health [71].

## 5. Conclusions

In conclusion, this study demonstrates that supplementing limiting amino acids (methionine, lysine, threonine) to diets with 0.7% reduced crude protein significantly enhances production performance in Hy-Line gray laying hens during their peak laying period, manifesting as higher egg production rates and lower feed conversion ratio. Furthermore, amino acid supplementation improved both immune function and nutrient total tract retention in laying hens. However, compared to the supplemented group, the 0.7% crude protein reduction alone exhibited minimal effects on most measured parameters. Notably, amino acid supplementation showed no significant impact on intestinal microbiota composition. These findings collectively indicate that amino acid supplementation elevates both productivity and health status in laying hens. Further research is warranted to identify specific amino acids within protein sources that modulate gut microbial communities.

## Figures and Tables

**Table 1:** Dietary composition and nutrient levels of the experimental diets.

Items	CON	NG	LAA
Ingredients, %			
Corn (7.8% crude protein)	62.3	62.3	62.3
Soybean meal (43% crude protein)	25.0	23.0	23.0
Limestone	9.00	9.00	8.92
Soybean oil	0.70	0.90	0.90
Wheat bran	-	1.80	1.80
L-Lysine (98%)	-	-	0.04
DL-Methionine (98%)	-	-	0.04
L-Threonine (98%)	-	-	0.03
Premix L33 ^1^	3.0	3.0	3.0
Total	100	100	100
Calculated nutrient levels ^2^			
Metabolic energy, (Kcal/kg)	2660	2660	2660
Crude protein, %	15.9	15.2	15.2
Digestible Lys, %	0.71	0.67	0.71
Digestible Met, %	0.38	0.37	0.41
Digestible Thr, %	0.49	0.46	0.49
Digestible sulfur-containing amino acids, %	0.58	0.57	0.61
Calcium, %	4.02	4.02	4.02
Phosphorus available, %	0.42	0.42	0.42

^1^ DSM Company L33 premix. Each kg of feed provides the following: vitamin A 9900 IU, vitamin D 34,000 IU, vitamin E 25 IU, vitamin K 32.5 mg, vitamin B12 mg, vitamin B26 mg, vitamin B64 mg, vitamin B12 0.024 mg, biotin 0.2 mg, pantothenic acid 10 mg, nicotinamide 35 mg, folic acid 1 mg, choline 360 mg, iron 80 mg, copper 10 mg, manganese 100 mg, zinc 100 mg, iodine 1.2 mg, selenium 0.3 mg, and methionine 1.5 g. ^2^ The crude protein is the measured value, the other nutritional components are calculated values.

**Table 2:** Effects of amino acid supplementation on production performance of laying hens under low-protein diet ^1^.

Items	CON	NG	LAA	SEM	*p*-Value
1–4 W					
ADFI, g/d	108.08	106.58	108.95	1.55	0.823
Average egg weight, g	56.80	55.73	56.74	0.25	0.208
FCR, g/g	1.90	1.91	1.92	0.02	0.936
Laying rate, %	93.63 ab	89.94 b	93.93 a	0.67	0.038
5–8 W					
ADFI, g/d	112.93	113.53	112.23	1.12	0.896
Average egg weight, g	58.79	57.72	58.65	0.22	0.155
FCR, g/g	1.92	1.97	1.91	0.01	0.245
Laying rate, %	96.79	95.00	96.31	0.39	0.205
9–12 W					
ADFI, g/d	114.30	116.2	114.16	0.68	0.426
Average egg weight, g	61.30	60.19	61.17	0.22	0.127
FCR, g/g	1.87 b	1.93 a	1.87 b	0.009	0.024
Laying rate, %	95.12	92.38	94.17	0.62	0.240
1–12 W					
ADFI, g/d	111.77	112.10	111.78	1.41	0.982
Average egg weight, g	58.96	57.88	58.86	0.59	0.368
FCR, g/g	1.90	1.94	1.90	0.02	0.120
Laying rate, %	95.18 a	92.44 b	94.80 a	0.70	0.018

^1^ Results are represented as Mean and SEM (n = 6). a,b means with different superscripts within the same row differ significantly, *p* < 0.05.

**Table 3:** Effects of amino acid supplementation on egg quality of laying hens ^1^.

Items	CON	NG	LAA	SEM	*p*-Value
Egg weight, g	62.40	60.16	61.21	0.73	0.110
Haugh unit	97.64 a	90.12 b	96.43 a	0.87	0.001
Eggshell strength, N	4.37	4.47	4.28	0.15	0.706
Eggshell thickness, mm	0.37	0.37	0.38	0.005	0.482
Egg shape index	1.30	1.31	1.31	0.001	0.902
Yolk rate, %	28.03	28.40	28.11	0.005	0.664

^1^ Results are represented as Mean and SEM (n = 6). a,b means with different superscripts within the same row differ significantly, *p* < 0.05.

**Table 4:** Effects of amino acid supplementation on serum biochemical parameters of laying hens ^1^.

Items	CON	NG	LAA	SEM	*p*-Value
TNF-α, ng/L	20.40	21.99	19.09	1.82	0.291
IGA, ng/mL	0.70	0.68	0.68	0.03	0.758
IGG, ng/mL	8.32	7.70	8.72	0.48	0.121
IGY, ng/mL	0.48 b	0.45 b	0.93 a	0.06	0.001
IL-6, ng/L	21.07	22.56	22.18	3.04	0.879
IL-10, ng/L	137.48 b	127.73 b	146.31 a	1.99	0.023

^1^ Results are represented as Mean and SEM (n = 6). a,b means with different superscripts within the same row differ significantly, *p* < 0.05.

**Table 5:** Effects of amino acid supplementation on total tract retention of laying hens (%) ^1^.

Items	CON	NG	LAA	SEM	*p*-Value
Crude protein (CP)	55.68 b	54.43 b	57.40 a	0.95	0.043
Ash	38.87	36.49	37.23	0.86	0.167
Calcium (Ca)	43.00	43.67	41.68	1.10	0.444
Phosphorus (P)	38.69 b	38.73 b	41.51 a	0.73	0.023

Retention data are provided as percentages. ^1^ Results are represented as Mean and SEM (n = 6). a,b means with different superscripts within the same row differ significantly, *p* < 0.05.

## Data Availability

The original contributions presented in this study are included in the article. Further inquiries can be directed to the corresponding author.
